# Chameleon Sequences—Structural Effects in Proteins Characterized by Hydrophobicity Disorder

**DOI:** 10.1021/acsomega.4c03658

**Published:** 2024-08-31

**Authors:** Irena Roterman, Mateusz Slupina, Katarzyna Stapor, Leszek Konieczny, Krzysztof Gądek, Piotr Nowakowski

**Affiliations:** † Department of Bioinformatics and Telemedicine, Jagiellonian University—Medical College, Medyczna 7, 30-688 Krakow, Poland; ‡ ALSTOM ZWUS Sp. z o.o., Modelarska 12, 40-142 Katowice, Poland; § Faculty of Automatic, Electronics and Computer Science, Department of Applied Informatics, Silesian University of Technology, Akademicka 16, 44-100 Gliwice, Poland; ∥ Chair of Medical Biochemistry, Jagiellonian University—Medical College, Kopernika 7, 31-034 Krakow, Poland; ⊥ AGH Cyfronet, SANO SCIENCE, Nawojki 11, 30-950 Kraków, Poland

## Abstract

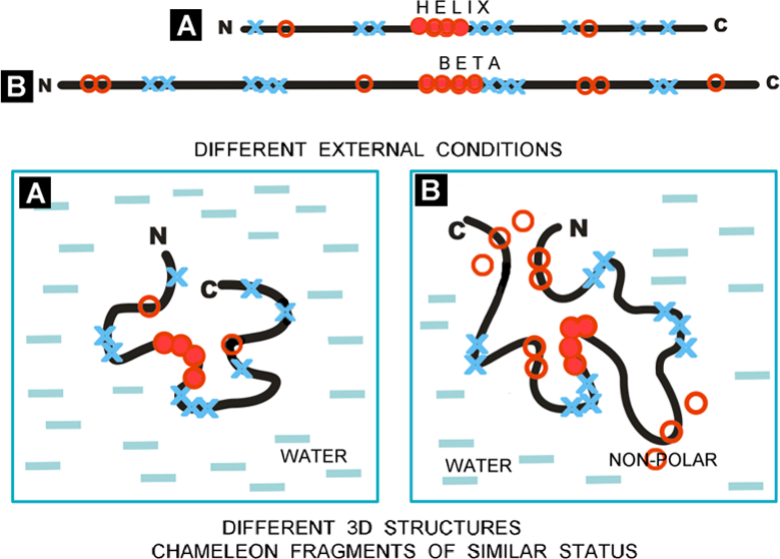

Repeated protein folding processes both in vivo and in vitro leading to the same structure for a specific amino acid sequence prove that the amino acid sequence determines protein structuring. This is also evidenced by the variability of structuring, dependent on the introduced mutations. An important phenomenon in this regard is the presence of a differentiated secondary structure for chain fragments of identical sequence representing distinct forms of the secondary-order structure. Proteins termed chameleon proteins contain polypeptide chain fragments of identical sequence (length 6–12 aa) showing structural differentiation: helix versus β-structure. In the present paper, it was shown that these fragments represent components matching the structural status dictated by the physicochemical properties of the entire structural unit. This structural matching is related to achieving the goal of the biological function of the structural unit. The corresponding secondary structure represents a means to achieving this goal, not an end in itself. A selected set of proteins from the ChSeq database have been analyzed using a fuzzy oil drop model (FOD-M) identifying the uniqueness of the hydrophobicity distribution taken as a medium for recording the specificity of a given protein and a given chameleon section in particular. It was shown that in the vast majority, the status of chameleon sections turns out to be comparable regardless of the represented secondary structure.

## Introduction

Proteins containing chameleon sequences have been the subject of numerous analyses revealing secondary structure variation for polypeptide chain sections with identical amino acid sequence.^1^ Studies identifying forms of secondary structure for a set of amino acids (4 aa) do not clearly indicate a well-defined preference.^2^ This issue is directly related to the basic assumption that a three-dimensional (3D) structure is determined by the amino acid sequence. Sections of identical sequences adopting a different form of secondary structure (helix versus β-structure) in different proteins prove that this relationship has its limitations and specificities.

Methods used to study this phenomenon include the technique of infinite-swap replica exchange incorporated into a molecular dynamics simulation. This technique has been identified as a convenient tool for fast structural sampling in changing conditions (temperature) to reveal the preferences for structure stabilization.^3,4^ Modifications in procedures oriented on protein folding simulation are focused on conformational space in search for energetically optimal organization with respect to secondary structure determination.^5,6^ A unique arrangement for β-hairpin generation has been defined as initiating ordering at early stages of folding.^7−9^

The present study concentrated on the contribution of chameleon sections to the overall structure of a structural unit (domain/single-chain protein) from the point of view of the hydrophobicity distribution present in the protein. It is assumed that protein folding in an aqueous environment aims to reproduce a micelle-like arrangement with a hydrophobic core in the central part of the molecule and a polar surface shell. However, it turns out that numerous proteins do not exhibit such a structure, which, based on the fuzzy oil drop model, is treated as a result of the contribution of an external environment different from polar water. Assessing the degree of dissimilarity of the hydrophobicity distribution in a structural unit against a micelle-like system allows the status of the entire structural unit to be assessed. It also becomes possible to assess the contribution of chameleon sections to the specific structure of the hydrophobic distribution of a given structural unit. In light of this analysis, it has been shown that the secondary structure adopted by the chameleon section is merely a form that supports the achievement of the superior goal of a specific hydrophobicity distribution in a given protein. The secondary structure of a chameleon section is therefore not an end in itself. It is the pursuit of a common structure that guarantees biological activity that influences the form of structuring of the chameleon section.

## Materials and Methods

### Description of the FOD-M Model Used

The analyses of the status of chameleon sections in relation to the hydrophobic core present in the protein molecule were performed using the fuzzy oil drop model (FOD-M) model.^10^ This model assumes that the distribution of hydrophobicity in a protein molecule can be assessed by two functions:

1. The observed distribution—referred here to as O_*i*_ —expressing the arrangement of interamino-acid hydrophobic interactions. The function introduced by Levitt^11^ was used here:
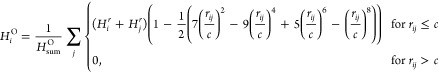
1)where *r_ij_* is the distance between the positions of the “effective atoms” (the averaged position of the atoms comprising a given amino acid) and *c* is the cutoff distance for these interactions taken according to ref (11) (9 Ǻ). The magnitude of interaction depends on the intrinsic hydrophobicity of interacting amino acids −*H^r^* (an arbitrary scale can be used^12^).
  The *H^o^* values (referred to as *O* in this paper) express the hydrophobicity level (following exactly the original paper^11^) present in the position of the given amino acid—the observed hydrophobicity level corresponding to the actual status of the given amino acid considering the immediate surroundings of the given amino acid.
2. The theoretical distribution—referred to as *T* herein (*T* stands for Theoretical, idealized)—is an idealized distribution fulfilling the criteria of a micelle-like system with a centrally located core and a gradually decreasing level of hydrophobicity up to zero at the surface. This distribution is expressed by a 3D Gaussian function spanning the protein body called *T* in this paper. The value of this function at the position of the effective atom expresses the expected idealized level of hydrophobicity (micelle-like distribution) at a given location of the protein.
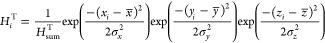
2)The values of the parameters σ_*x*_, σ_*y*_, and σ_*z*_ are matched to the size and shape of the studied protein, expressing its size and shape.
  The *H*^T^ value expresses an idealized, theoretical level of hydrophobicity (hereinafter referred to as *T*), assuming a micelle-like distribution.
  The use of this function calls for uniform orientation of proteins for which the 3D Gaussian is plotted. This orientation is defined as follows: 1. Geometric center located at the origin of the coordinate system (0,0,0); 2. Distances between each pair of atoms comprising the molecule (*r_ij_*) are calculated, and the pair with the greatest distance is selected. Then, the entire molecule is oriented such that the line between this pair of atoms coincides with the *X* axis; 3. Lines connecting all pairs of atoms are projected onto the *YZ* plane, and the longest such line is selected. Then, the molecule is rotated so that this line coincides with the *Y* axis; 4. Values of σ_*x*_, σ_*y*_, and σ_*z*_ are computed (according to the three-sigma rule) for the resulting orientation; 5. Locations of effective atoms (averaged-out positions of all atoms comprising the given amino acid residue) are computed (as *x_i_*, *y_i_*, and *z_i_*, respectively). The value of the 3D Gaussian at each point is then assumed to correspond to local hydrophobicity in an ideal micelle-like structure. This reference distribution is denoted *T*.

The comparison of these distributions allows an assessment of the degree of match between distribution *O* and distribution *T*. The comparison is possible after normalizing both distributions (first positions in eqs 1 and 2). Quantitatively, this assessment is carried out using the divergence entropy introduced by Kullback–Leibler.^13^
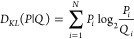
3where the *P* distribution—the distribution examined—in the case of the FOD model is the *O* distribution, the *Q* distribution is the reference distribution, and in the FOD model—the *T* distribution. The determined *D*_KL_ value for the relationship (*O|T*) is not interpretable. Therefore, a second reference distribution—called here *R* distribution (*R* stands from Random–each effective atom represents the equal probability to be hydrophobic)—is introduced with *R*_*i*_*=* 1/*N*, where *N* is the number of amino acids in the chain. This is a unified distribution for the whole molecule, where each amino acid is assigned a constant equal to the *R*_*i*_ value. The *D*_KL_ value for the (*O|R*) relationship determines the degree of closeness of the *O* distribution to a distribution devoid of a hydrophobic core (uniform distribution throughout the protein).

The *D*_KL_*(O|T) < D*_KL_*(O|R)* relationship is interpreted as stating the presence of a hydrophobic core. The degree of fit of the *O* distribution to the *T* distribution is expressed by the *RD* (Relative Distance) parameter determined according to the equation:
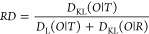
4A value of *RD* < 0.5 is interpreted as the presence of a hydrophobic core.

In the analysis so far, proteins with a low *RD* value have been identified, which provides evidence for the correctness of the proposed model. These proteins are downhill, fast-folding, ultrafast-folding, and antifreeze type II proteins.^14^

It should be noted that the model in question can be applied to any defined structural unit: a multichain complex, a single chain, or a domain. A 3D Gaussian function may be generated for each listed structural unit. It is also possible to determine the status of a selected chain section and its importance (role) in the structure of the distribution present in a given structural unit. An *RD* value < 0.5 for a selected fragment (this can be the status of a single chain in the complex or the status of a chain fragment in relation to an arbitrarily selected structural unit) indicates the participation of this fragment in building a unit-wide type of hydrophobicity distribution as locally fulfilling micelle-like distribution conditions. A value of *RD* > 0.5 for a selected chain fragment indicates its local mismatch with the hydrophobic core structure within the entire structural unit. When analyzing the status of a selected structural unit fragment, the set of *O*_*i*_, *T*_*i*_, and *R*_*i*_ values is subjected to a normalization procedure.

**Figure 1:**
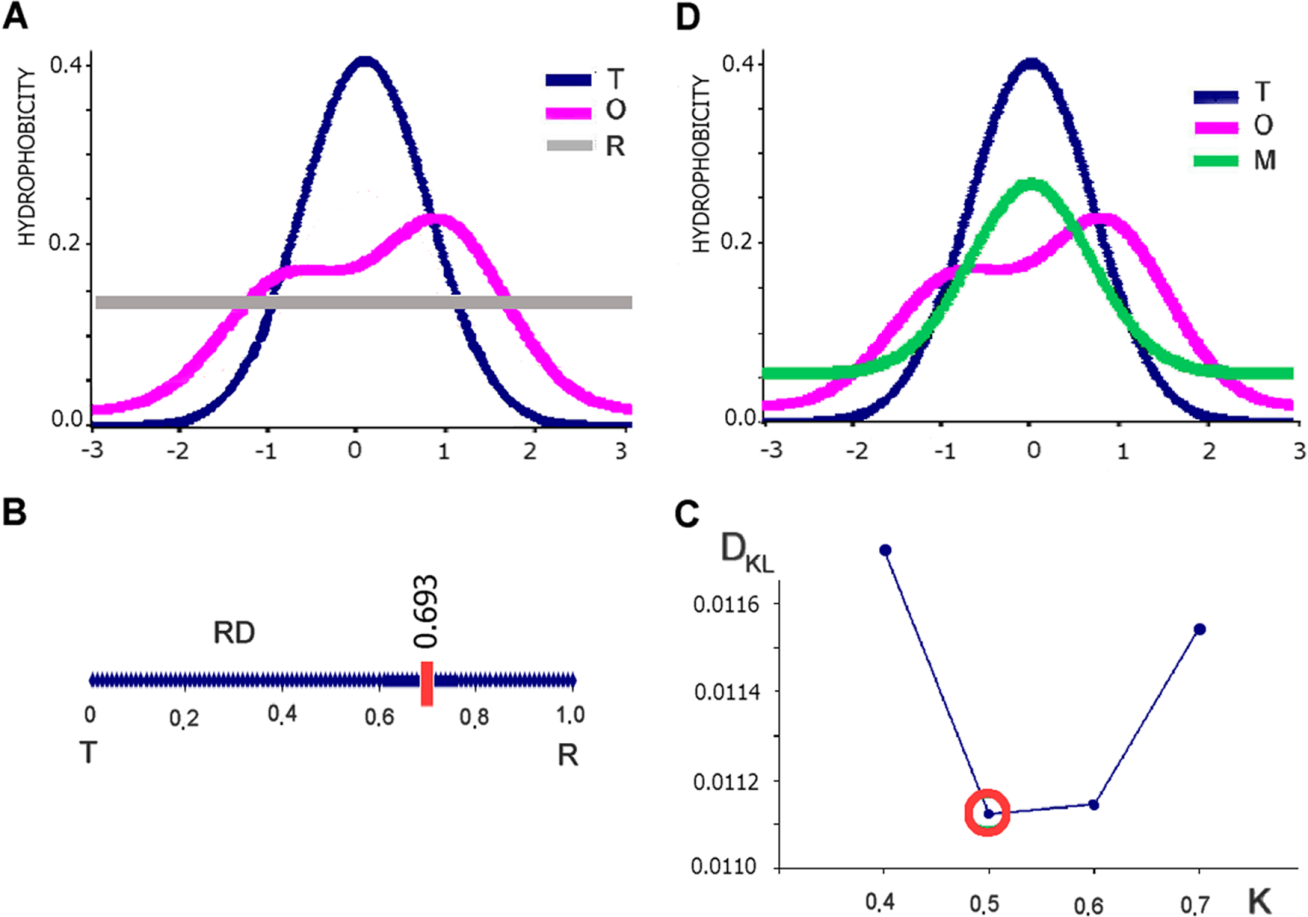
Visualization of the model used: (A) example of *T* (blue), *O* (pink), and *R* (gray) distribution form reduced to 1D; (B) determined value of parameter *RD* = 0.693, suggesting the status of hydrophobicity distribution close to *R*; (C) method of determining the value of the *K* parameter—the value corresponds to the minimum *D*_KL_ value for the (*O|M*) relationship; (D) summary of *T* (blue), *O* (pink), and *M* (green) distributions for the *K* value determined as in (C).

This part of the model is visualized in Figure 1A, where the *O* (pink) distribution is compared with the reference distribution *T* (blue—hydrophobic core present) and with the reference distribution *R* (green). The determined value *RD* = 0.693 for the given example is given on the axis of variation of *RD* values (Figure 1B). It suggests treating the *O* distribution as not meeting the micelle-like criterion.

The model discussed herein has been used to assess the status of a structural unit where a chameleon fragment is present and the status of the section itself with an identical sequence in two different proteins. The structural units used in the present analysis follow the CATH classification.^15^ The status of the chameleon sections is determined relative to the structural unit in which the section is present.

The modified FOD-M model takes into account the presence of a nonaqueous (or modified aqueous) environment for a given protein, which is assumed to have influenced the folding process of the protein not necessarily directing the structuring toward the generation of a centric hydrophobic core. The degree of mismatch between the distribution *O* and the distribution *T* may be due to the different characteristics of the environment, which, by actively participating in the protein folding process, may direct the folding process toward a distribution different than micelle-like. The construction of a centric core with a polar shell is characteristic of an aqueous environment interacting with bipolar molecules, which is what all amino acids are (with a variable ratio of polar to hydrophobic parts).

The presence and influence of an external field on the formation of the hydrophobicity distribution within a protein molecule have been defined, starting from the characteristics of membrane proteins, where, for a favorable hydrophobic interaction between a part of the membrane and the anchored protein, the exposure of hydrophobic amino acids on the protein surface is expected. In addition, especially for membrane proteins acting as an ion channel, a concentration of polar residues in the central part of the protein is also expected. Therefore, the description of the force field in a membrane protein is expressed by a function complementary to the Gaussian function:
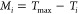
5

In fact, the analysis of membrane proteins suggests the use of the following equation to record the force field based on hydrophobicity
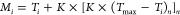
6In this field record, there is a water-derived field (3D Gauss function indicated as *T*_*i*_) modified by the presence of a complement field (*T*_max_*– T*_*i*_) to a degree expressed by the value of the parameter *K*. The parameter *K* can take the value *K* = 0.0 for proteins with a hydrophobicity structure compatible with a centric core and a polar surface up to a value *K* > 3 for proteins showing structuring far from the micelle-like form (including certain membrane proteins).

The value of the *K* parameter is determined as part of the procedure to find the minimum value of *D*_KL_*(O|M)* (Figure 1C), assuming that the value of the *K* parameter thus determined defines the *M* distribution most similar to the *O* distribution. Thus, it can also indicate the degree of dissimilarity (with respect to the polar water field) of the external field for protein folding (Figure 1D).

The interpretation of the FOD-M model parameters is as follows:

1. The *RD* value indicates the extent to which the micelle-like system has been reproduced in the protein structure. High *RD* values indicate a significant dissimilarity of the distribution to the micelle-like distribution with a centric hydrophobic core. It also means an approximation of the *O* distribution to the uniform *R* distribution, lacking the central nature of hydrophobicity in the protein.
2. The *K* value determines the degree to which factors alter the specificity of the aqueous environment by introducing a hydrophobic factor in particular. The higher the *K* value, the weaker the influence of the polar water environment on the structuring directed toward the generation of the hydrophobic core.

The presented analysis focuses on assessing the role of chameleon fragments in the overall distribution of hydrophobicity. For this reason, we will now focus on calculating values of *RD*_FR_, where the _FR_ index identifies the given fragment.

Selected fragments of *T*, *O*, and *R* profiles (in this case, corresponding to the chameleon fragment) should be normalized to enable meaningful comparisons. The calculated value of *RD*_FR_ determines the influence of the given fragment upon the overall distribution of hydrophobicity within the protein molecule as a whole. If the fragment contributes to the formation of a hydrophobic core, we observe *RD*_FR_*<* 0.5; otherwise, we regard it as locally divergent from the theoretical (idealized) distribution. The same operation can be performed for protein dimers in order to determine whether the given fragment participates in the formation of a shared hydrophobic core.

Applying the FOD-M model to assess the status of chameleon fragments consists primarily of determining the properties of the given structural units using *RD* and *K* parameters, followed by analysis of the chameleon fragment itself. The extent to which it participates in forming a hydrophobic core depends on the computed value of *RD*_FR_. We then investigate the relation between the hydrophobicity status and the secondary conformation of the fragment in the context of the entire structural unit (protein or domain).

### Data

The ChSeq database available at prodata.swmed.edu/chseq was used as the source for pairs of proteins with chameleon sequences representing different: helical and β-structural forms for chameleon sequence fragments.^16,17^ The structure of the proteins listed in the ChSeq database was taken from the PDB database.^18^

Due to the very large number of chameleon protein pairs in the ChSeq database,^16,17^ the present study analyzed a sub-base.

The presented proteins have been selected on the basis of the following criteria: all protein pairs contained in the ChSeq database with a stringent length criterion of 7 (777 structures in total) were processed using the FOD-M model with *RD* and *K* values calculated for each protein (or domain, as appropriate).

The above-mentioned data set was divided into three categories:

1. Both proteins belonging to the chameleon pair exhibiting *RD* < 0.5^19^
2. Both proteins belonging to the chameleon pair exhibiting *RD* > 0.5 (object of this publication)
3. One protein exhibiting *RD* < 0.5 with its partner exhibiting *RD* > 0.5^20^

Each group was then subjected to an independent analysis. The presented work focuses on category 2, which comprises 293 protein structures, all of which were taken into account in the discussion of the Results section.

Given that ChSeq does not include a search tool, Table 1 lists the entry IDs of each analyzed protein in the database.

**Table 1:** Characteristics of Pairs of Proteins Discussed in Detail in the Present Work^a^

β-structure				helix		
PDB ID	function	source organism	sequence	source organism	function	PDB ID
(1)—1J93^21^	lyase	plant	AVLGFVG	bacteria (thermo)	hydrolase	2Z1K(22)
(2)—3M5U^23^	transferase	bacteria	IALFYGG	ecoli	transport	2NPD(24)
(3)—1K4Y^25^	hydrolase	rabbit	VAVFLGV	wild pig	membrane prot	4HYT(26)
(4)—1Q7G^27^	oxidoreductase	baker’s yeast	YNLVLLA	human	signaling	2LNL(28)
(5)—2COI^29^	transferase	human	ALLFVLL	bacteria	transport	3Q7K(30)
(6)—1MVM^31^	DNA binding	virus	ALATRLV	fungi	protein bind.	3RMR(32)
(7)—2EBE^33^	unknown	bacteria (thermo)	GRFYQLT	artificial	steroid bind.	3RY9(34)
(8)—2D4Z^35^	transport	fish	DTNTLLGS	fungi	hydrolase	1GAH-A(36)

a Selected pairs of proteins are indicated in Figure 3C (green lines). The left column also gives the positions as indicated in Figure 3C. To facilitate identification of selected pairs of proteins, the following numbers on the list in ChSeq database are given: (1)—position 727, (2)—242, (3)—113, (4)—208, (5)—503, (6)—7, (7)—342, (8)—the only one example not present in ChSeq—postions of residues in 2D4Z—578–585 and 245–252 in 1GAH.

Sections corresponding to chameleon fragments were extracted from *T*, *O*, and *R* profiles and normalized to enable calculation of *RD*_FR_ values, with the FR index referring to “fragment”. These values determine the local status of the fragment under analysis, as well as its contribution to the overall distribution of hydrophobicity within the protein molecule. The role of the fragment may be to support construction of an overall hydrophobic core (*RD*_FR_*<* 0.5) or to obstruct it by introducing local disorder (*RD*_FR_*>* 0.5).

Results were analyzed in the form of a scatter diagram visualizing the relationship between the status (expressed by *RD* value) of the structural units (chain, domain) in the protein pairs and the relationship of the status expressed by *RD*_FR_ of chameleon sections. The determined correlation coefficient for the distribution of status-representing dots (*RD*_FR_) in the chameleon sections was taken as an indicator of the status match between these sections, regardless of their secondary structure.

Detailed characteristics are given only for selected proteins (selection criterion given in the Results section, Figure 3C). The characteristics of the proteins discussed in detail are listed in Table 1.

### Analysis of the Status of Chameleon Sections

All proteins analyzed here are characterized according to the following pattern:

1. A structural unit is identified within which a chameleon fragment is present (according to the CATH classification^15^) available in the PDBSum database.^37^
2. For an identified structural unit (chain or domain), the *RD* value is determined to determine the extent to which the structural unit reproduces the micelle-like system.
3. For this unit, the *K* value is also determined to identify the degree of dissimilarity of the environment affecting the structuring in relation to an external force field such as polar water.^38,39^
4. For the chameleon section, the *RD*_FR_ value is determined to identify the form of the local contribution of the given fragment in relation to the hydrophobicity distribution within the structural unit.

These results are assessed on the basis of the correlation coefficient, based on *RD*_FR_ values—relating the status of helical and β folds. High values of the correlation coefficient indicate that chameleon fragments perform a similar role in determining the structure of the overall hydrophobic core.

To visualize the model used, an abstract example of the influence of the environment on the formation of the structure with varied secondary structure for a chameleon section is used (Figure 3).

**Figure 2:**
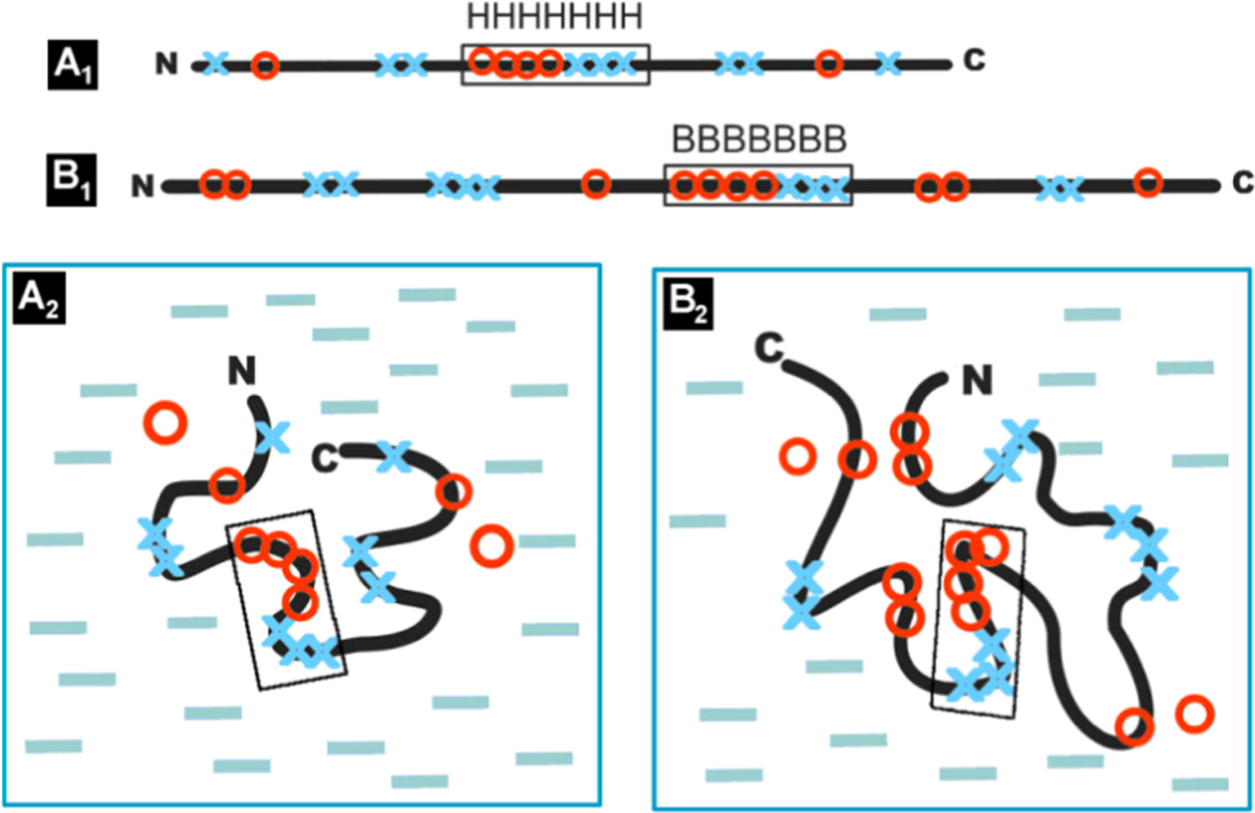
Summary of the influence of the specific environment on the structuring of a polypeptide chain with a hydrophobicity (red O)/polarity (blue X) distribution of amino acids (aa sequences with varied hydrophobicity), top line with a highlighted different secondary structure for chameleon sections (boxes). A_1_ Sequence with polar (blue X) and hydrophobic (red O) residues distinguished. Sequence in frame—the chameleon sequence in helical form. A_2_—the chain (as in A_1_) folded in the environment (blue dash—water, red circle—hydrophobic factor)—the effect of environment with varying polarity on the structuring of the sequence listed in the upper row on top. B_1_ Sequence with polar (blue X) and hydrophobic (red O) residues distinguished. Sequence in frame—the chameleon sequence in β-structural form. B_2_—the chain (as in B_1_) folded in the environment (blue dash—water, red circle—hydrophobic factor)—the effect of environment with varying polarity on the structuring of the sequence listed in the upper row on top.

Figure 2 is intended to introduce a justification needed for the variation in the effects of polypeptide chain folding depending on the environment.

The identification of the different status of the chameleon sections (expressed by the *RD* parameters)—as assumed in the FOD-M model—is precisely due to the influence of the environment and not only the characteristics of the amino acids themselves included in the compared sections with identical sequence.

In both of the examples presented, the essence lies in adjusting the status of the polypeptide chain section in relation to the specificity of the local environment during folding. Obtaining a status within the external force field that is compatible with it can guarantee a suitable form of the secondary structure. Secondary structure is thus a means to an end and not an end in itself.

### Programs Used

The potential used has two possible accesses to the program:

The program allowing calculation of *RD* is accessible upon request on CodeOcean platform: https://codeocean.com/capsule/3084411/tree. Please contact the corresponding author to get access to your private program instance.

The application—implemented in collaboration with the Sano Center for Computational Medicine (https://sano.science) and running on resources contributed by ACC Cyfronet AGH (https://www.cyfronet.pl) in the framework of the PL-Grid Infrastructure (https://plgrid.pl)—provides a web wrapper for the above-mentioned computational component and is freely available at https://hphob.sano.science.

The VMD program was used to present the 3D structures.^40,41^

## Results

In the entire set of proteins analyzed (ChSeq database), 293 part proteins fulfilling the *RD* > 0.5 condition in both proteins constituting a pair representing the helical structure and β-structure for the chameleon sections were identified.

**Figure 3:**
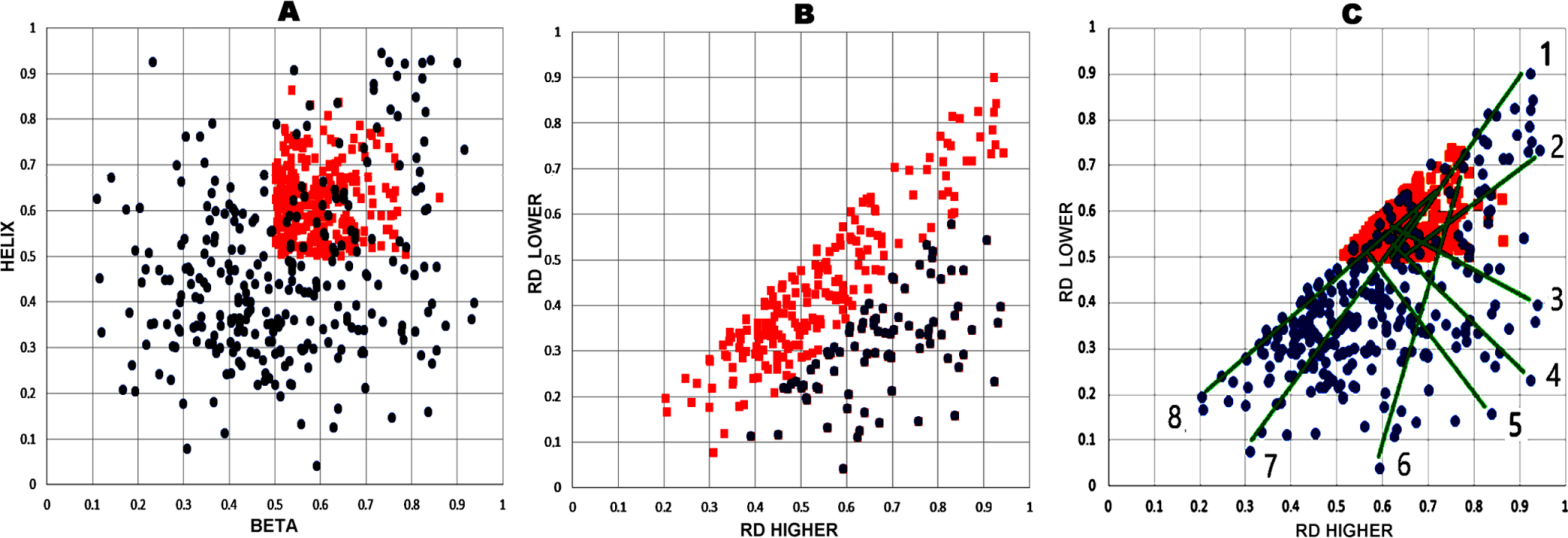
Characteristics of a set of proteins with status expressed as *RD* > 0.5. (A) Distribution of *RD* values in pairs of chameleon proteins; red dots: status of structural units (domain/chain), blue dots: status of chameleon sections for the status relationships for β-structural sections (horizontal axis) and helical sections (vertical axis). Correlation coefficient for dots representing the status of chameleon sections = 0.571. (B) Distribution of *RD*_FR_ values expressing the status of chameleon sections in pairs of chameleon proteins ordered by arrangement; horizontal axis—*RD*_FR_ values higher relative to lower *RD*_FR_ values (vertical axis), for the complete set; the correlation coefficient for the status relationship in the ordered arrangement (axis system) after elimination of outliers (blue dots) = 0.91. This value indicates the status of 70% of protein pairs in the analyzed database. (C) Arrangement of the relationships (green line) of the status of the structural units (domain/protein) against the status of the chameleon sections present in them. Those positions that show extreme *RD*_FR_ values in chameleon section pairs in relation to the status of the structural units are highlighted. These positions are analyzed in detail later in the paper.

Figure 3A visualizes the status of the subgroup analyzed in this work. The collection of red points reveals the status of protein pairs selected for analysis. According to the selection criteria, *RD* values for both structural units should be in excess of 0.5 (red dots). At the same time, the variation in the status of chameleon fragments is shown in blue. Figure 3A therefore groups protein pairs and chameleon fragments by their secondary structural characteristics (horizontal axis, β folds; vertical axis, helical folds). Our goal is not to determine the relations between these types of secondary folds, but rather to compare the status of both fragments regardless of their conformational properties, and to determine whether both structural forms contribute in a similar way to the resulting hydrophobicity distribution. Therefore, the set described by the *RD* values for chameleon sections was ordered, revealing the relationship: on the horizontal axis, the *RD* values were higher, on the vertical axis, the *RD* values were lower regardless of the secondary form of the chameleon fragment. For such an ordered base, the relationship of the status of chameleon fragment using_RDFR_ is expressed with a correlation coefficient = 0.571 (Figure 3B, all dots). Such values of the correlation coefficient determine the correspondence between the status of various chameleon fragments in the input data set, with high values of the coefficient indicating relatively good alignment between the ways in which each structural form contributes to the overall distribution of hydrophobicity. In the scatterplot shown in Figure 3B (all dots—*RD*_FR_ values), outlier dots were subjectively highlighted (Figure 3B, blue dots), thus identifying those pairs of chameleon sections where the status of the sections is comparable in both forms of secondary structure (Figure 3B, red dots).

The elimination of outlier dots (blue dots in Figure 3B, outlier dot elimination was carried out stepwise until a correlation coefficient >0.9 was reached) representing 30% of the analyzed pairs allows the evaluation of the remaining pairs of proteins representing a highly comparable status of chameleon sections (irrespective of secondary structure form), showing a correlation coefficient (Figure 3B, red dots) of 0.91. This value indicates a highly comparable status of sections differing essentially in terms of secondary structure.

The portion that comprises 70% of the base confirms the hypothesis regarding the comparable contribution of chameleon sections to the structure of the whole structural unit regardless of secondary form. In contrast, structures represented by outlier dots call for further analysis of the reasons behind their unusual characteristics. Figure 3C once again visualizes the relation expressed by *RD*_FR_ (horizontal axis—higher values of *RD*_FR_; vertical axis—lower values of *RD*_FR_ in pairs). The analyzed subgroup (structural units with *RD* > 0.5) is further highlighted using red dots in Figure 3C. Extreme outliers (in terms of *RD*_FR_) are also visualized. Some of them (3–6) strongly diverge in terms of status, whereas in others (1 and 8) both chameleon fragments share a similar status. In the protein pair numbered 1, these fragments contribute in a very similar way to the structure of the hydrophobic core, despite exhibiting very strong local discordance versus the idealized micelle-like structure. A similar situation occurs in the case of the pair of chameleon fragments numbered 8, both of which are strongly accordant and conform locally to the micelle-like distribution, despite the fact that the proteins themselves do not exhibit micellar characteristics (*RD* > 0.5). The same considerations apply to pairs numbered 2 and 7.

The characteristics of the proteins highlighted in Figure 3C are given in Table 1. The status of selected examples discussed in detail later in this work is given in Table 2, where the *RD* parameter values for the structural units and the status of the chameleon sections as components of the structures of these units are provided. The sequence of the chameleon sections is also given. In addition to the *RD* values to determine the status of the domains/chains, the *K* coefficient value is also given, which, according to the interpretation of the FOD-M model, determines the contribution of the nonaqueous factor in shaping the structure of the structural unit.

**Table 2:** Characteristics of the Protein Pairs Highlighted in Figure 3C Using Green Lines

β				helix		
PDB ID	*RD*/*K* domain	*RD*_FR_	sequence	*RD*_FR_	*RD*/*K* domain	PDB ID
1—1J93	0.545/0.55	0.923	AVLGFVG	0.901	0.638/0.85	2Z1K
2—3M5U	0.516/0.55	0.944	IALFYGG	0.735	0.637/0.70	2NPD
3—1K4Y	0.566/0.60	0.992	VAVFLGV	0.361	0.612/0.57	4HYT
4—1Q7G	0.502/0.43	0.924	YNLVLLA	0.233	0.677/0.69	2LNL
5—2COI	0.668/0.94	0.837	ALLFVLL	0.158	0.571/0.53	3Q7K
6—1MVM	0.764/1.16	0.593	ALATRLV	0.040	0.642/0.71	3RMR
7—2EBE	0.508/0.42	0.308	GRFYQLT	0.078	0.571/0.61	3RY9
8—2D4Z	0.670/0.78	0.204	DTNTLLGS	0.195	0.734/1.19	1GAH-A

It should be noted that the chameleon proteins selected in the present sub-base represent a status with *RD* > 0.5, which means (according to the interpretation of the model used) that their folding took place with the participation of a nonaqueous environment. In particular, a targeting factor could be the presence of **“**chaperone” proteins,^38^ the cell membrane^10^ or another system affecting the reduction of the external force field in the form of polar water.

The conditions for changing the environment can theoretically be very different. Theoretically, therefore, a comparable status of chameleon sections was not expected, as their structuring may also be the result of the contribution of specific environmental factors. The high value of correlation coefficient obtained for 70% of the representatives of the analyzed group indicates a significantly comparable status of the chameleon sections regardless of the secondary structure they represent.

### Detailed Analysis of Chameleon Proteins with Extreme Status of Chameleon Sections (Figure 3C)

Chameleon sections representing dots highlighted as extreme outliers with respect to the *RD*_FR_(helix) ≈ *RD*_FR_(β-structure) relationship are analyzed in the context of searching for the reasons for the exceptional status of the discussed proteins. To this end, *T*, *O*, and *M* profiles for the entire structural unit are presented with the location of the chameleon section given. 3D structures with highlighted chameleon sections are also provided. The positions of catalytic residues were also included as a link to the biological activity record of the protein for which it was being looked for. To visualize the status of chameleon sections themselves, their *T* and *O* profiles are also presented, revealing the reasons for the respective *RD*_FR_ parameter values describing them.

#### Chameleon Sections Representing Similar Status Representing Extremely High *RD*_FR_ Values

**Figure 4:**
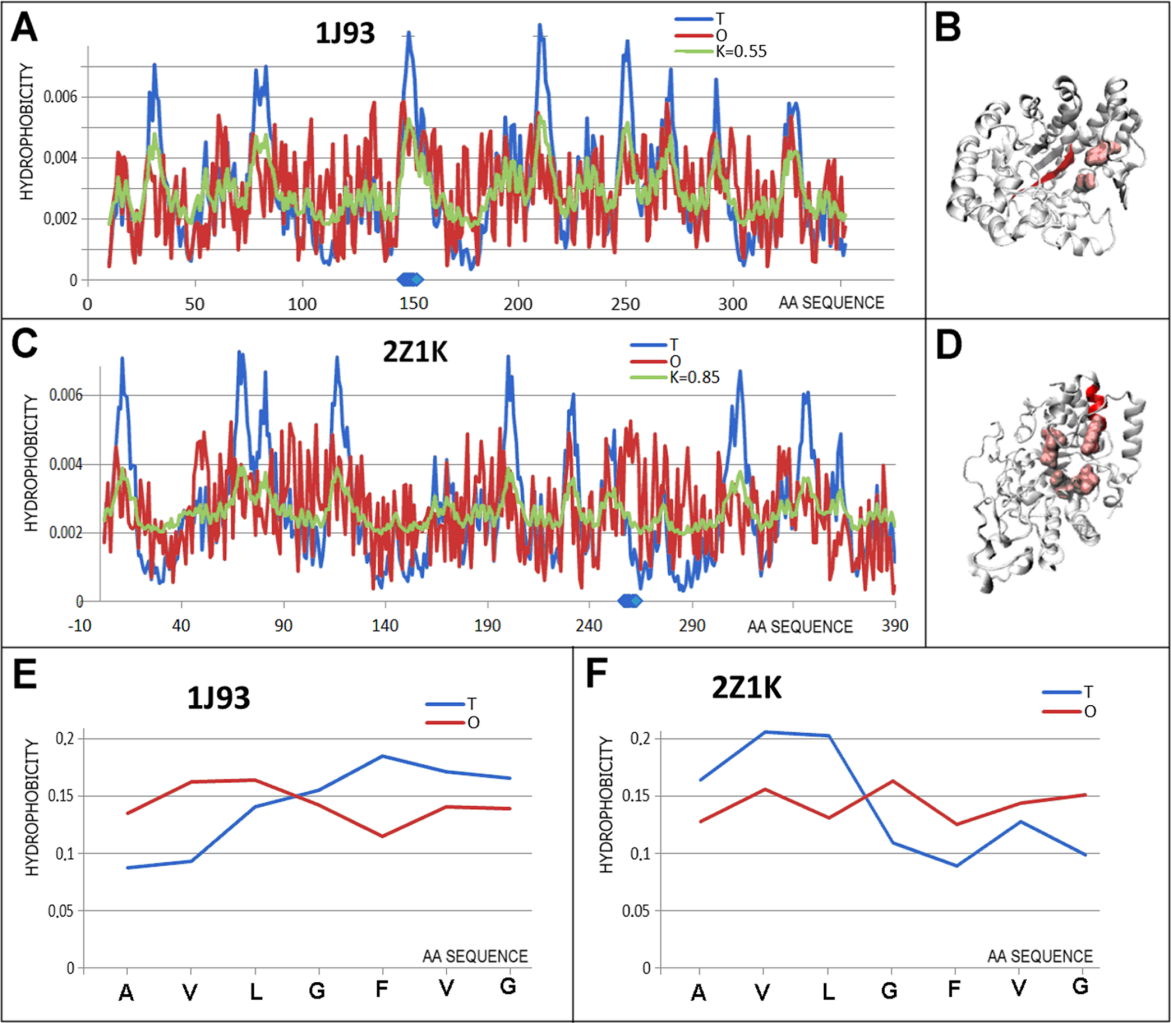
Pair characterization denoted as 1 in Figure 3C. (A) *T*, *O*, and *M* distribution for the *K* value (given in the legend), describing the status of lyase (1J93). (B) 3D representation of the structural unit for which the parameters have been determined with the distinguished chameleon section (red) highlighted—1J93Z, highlighted residues (pink)—catalytic residues. (C) *T*, *O*, and *M* distribution for the *K* value (given in the legend), describing the status of hydrolase (2Z1K). (D) 3D representation of the structural unit for which the parameters have been determined with the chameleon section (red) highlighted—32Z1K. Additionally, the positions of the catalytic residues are highlighted in pink. (E) *T* and *O* distribution for the chameleon section in lyase (1J93). (F) Set of *T* and *O* distributions for the chameleon section in hydrolase (2Z1K). Blue dots on *X*-axis in (A, C)—chameleon fragment.

The first highlighted pair of chameleon proteins is representative of structural units with a status relatively slightly above the accepted limit value (*RD* = 0.5). This means that the entire structural unit (single-chain in the case of 1J93—enzyme lyase and domain in the case of 2Z1K—hydrolase) represents an ordering relatively close to a micelle-like arrangement. This is indicated by the value *K* = 0.55 in the case of 1J93. In contrast, the 2Z1K domain (2–390 aa) shows an arrangement far from a micelle-like ordering (Figure 3C, dot 1). The relatively high value *K* = 0.85 for 2Z1K suggests a significant contribution of nonaqueous factors to the structuring of this protein. The 2Z1K protein is a thermophilic bacterial protein, which implies significantly different environmental conditions from those in the aqueous environment under standard thermal conditions. Nevertheless, despite this structural variability, the status of chameleon sections reveals significant similarity expressed by extremely high *RD*_FR_ values. This means that a local task for chameleon sections in both proteins of different activity and biological origin is expected to fulfill a role realized by a hydrophobicity distribution highly different from a micelle-like arrangement. The comparable status of the two chameleon sections expressed as a set of *T* and *O* profiles (Figure 4C,D) reveals the reasons for this high *RD*_FR_ value. In both cases, the O distribution clearly contrasts with the arrangement expected by idealized distribution. This consistency of local status for comparable sections with identical sequence but different secondary structure seems to fulfill the expectation for providing aim-oriented activity obtained by means of a different secondary structure but with a common status toward the whole protein/domain. In both cases, the chameleon sections are located in close proximity to the catalytic center. In the case of 1J93, the central location of the chameleon section suggests reduced stability of the hydrophobic core. It might be expected that this section probably also provides a suitable local external field for the catalytic reaction itself. The role of mismatching the hydrophobicity distribution against the idealized distribution in the case of 2Z1K located on the protein surface in the immediate vicinity of the catalytic center is most probably critical for the interaction with the substrate. Providing the right conditions of the local section of the external force field is important for the interaction leading to a highly specific catalytic reaction (Figure 4).

#### Example of Proteins with Chameleon Sections Representing Increasing Divergence of the Status Match for Both Chameleon Sections (Dot 2—Figure 3C)

**Figure 5:**
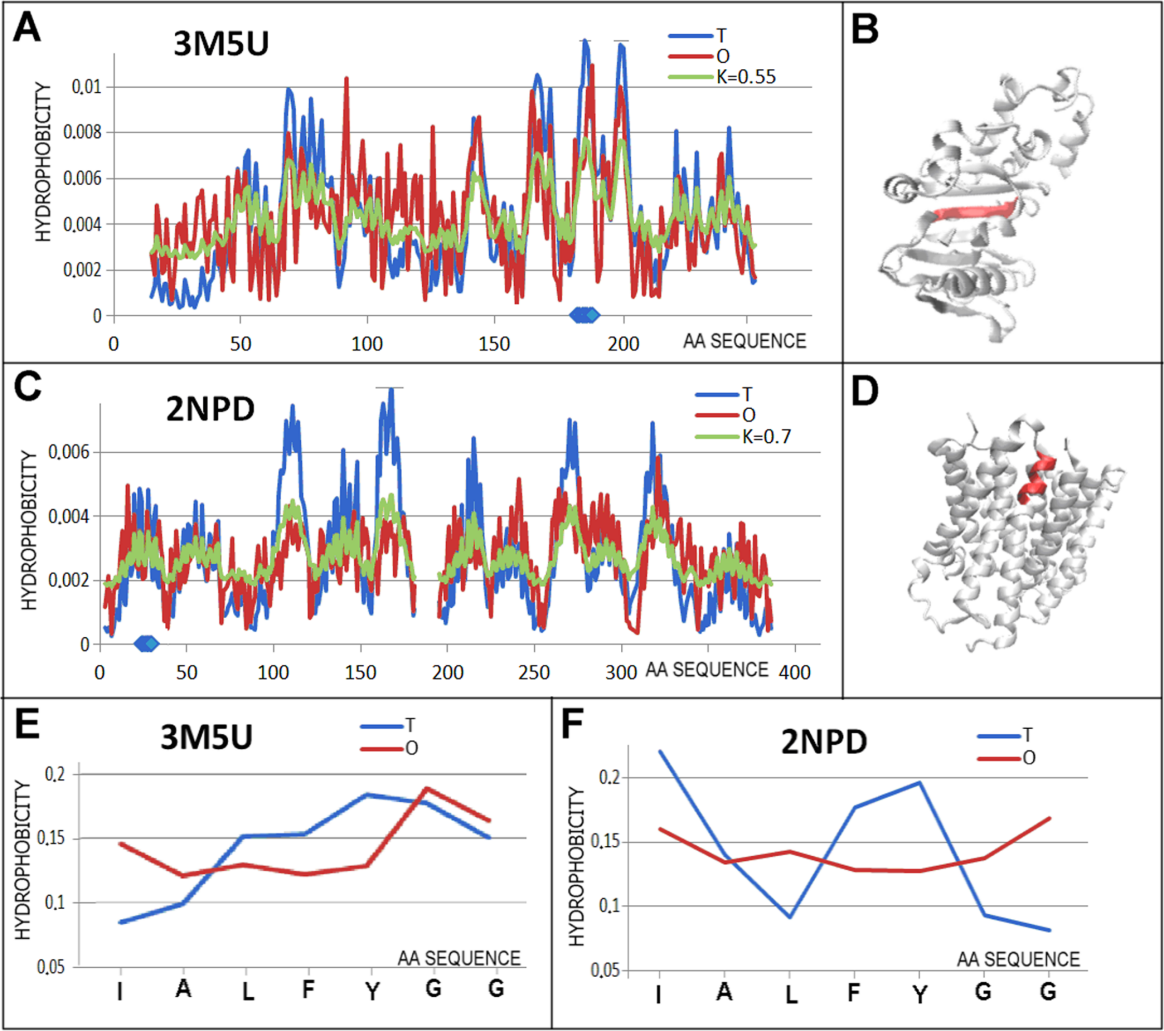
Pair characterization distinguished as 2 in Figure 3C. (A) *T, O*, and *M* distribution for the *K* value (given in the legend), describing the status of transferase (3M5U). (B) 3D representation of the structural unit for which the parameters have been determined with the chameleon section (red) highlighted −3M5U. (C) *T, O*, and *M* distribution for the *K* value (given in the legend) describing the status of protein engaged in transport (2NPD). (D) 3D representation of the structural unit for which the parameters have been determined with the chameleon section (red) highlighted −2NPD. (E) *T* and *O* distribution for the chameleon section in transferase (3M5U). (F) Set of *T* and *O* distributions for the chameleon section in protein engaged in transport (2NPD). Blue dots on *X*-axis in (A, C)—chameleon fragment.

A similar arrangement is present in a pair of chameleon proteins: phosphoserine aminotransferase (3M5U, domain 15–253) and ammonia channel (2NPD, single complete chain) (Figure 5A,B). The status of chameleon sections is very high in both cases mentioned (*RD*_FR_ = 0.944 and *RD*_FR_ = 0.735, respectively). The position of the dot representing this status deviates from the line expressing equal status values for both compared sections, this pair can be classified as examples with comparable chameleon section status. Its uniqueness is due to the extremely high mismatch of the *O* distribution with the *T* distribution identified in the 3M5U protein (seen in Figure 5D). Using a qualitative assessment, it can be concluded that the status of the proteins forming the chameleon pair analyzed is comparably highly mismatched with the distribution expected for a micelle-like arrangement.

The interpretation of the arrangement of the *T* and *O* distributions in the chameleon section in aminotransferase located in the central part of the molecule may suggest the introduction of local instability, as the central location indicates the involvement in the structure of the hydrophobic core. The arrangement of the *O* and *T* levels (Figure 5C) indicates a divergence of the levels. The chameleon section in the protein responsible for transport (membrane protein) is located in the area of change of contact with the polar environment (loops) and the part of the protein remaining in contact with the membrane. The large variability in the *T* levels in this section—specifically in the helix arrangement—shows the need of contact with the surrounding water (low *T* levels) while interacting with the hydrophobic membrane (high Ti levels). The expected *O_i_* levels do not follow such changes. The almost constant status of the chameleon section may introduce a certain local instability, which, with proteins playing a channel role, is perhaps important for the function performed.

#### Status with Extremely High Mismatch with the Micelle-like Arrangement versus Status Close to the Micelle-like Arrangement (Dots 3, 4, and 5—Figure 3C)

Significant variation in the status of chameleon sections was identified in the pairs: 1K4Y and 4HYT, 1Q7G and 2LNL, and 2COI and 3Q7K. The pairs of proteins discussed are characterized by very high *RD*_FR_ values for the chameleon (β-form) sections with decreasing *RD*_FR_ levels for the helical form. These extremely high *RD*_FR_ values are above and close to the 0.9 level, which is very rarely observed in the analysis of different proteins to date.

**Figure 6:**
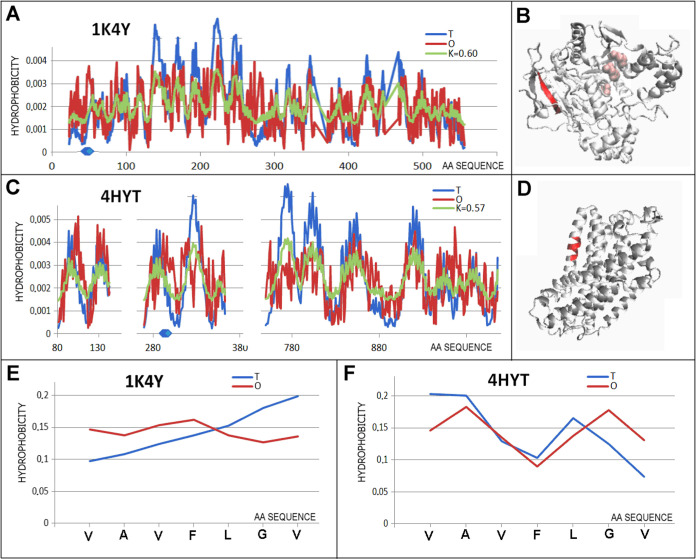
Characteristics of the pair distinguished as 3 in Figure 3C. (A) *T, O*, and *M* distribution for the *K* value (given in the legend) describing the status of hydrolase (1K4Y). (B) 3D representation of the structural unit for which the parameters have been determined with the distinguished chameleon section (red) highlighted—1K4Y, highlighted residues as pink—catalytic residues. (C) *T, O*, and *M* distribution for the *K* value (given in the legend) describing the status of membrane protein (4HYT). (D) 3D representation of the structural unit for which the parameters have been determined with the chameleon section (red) highlighted—4HYT. (E) *T* and *O* distribution for the chameleon section in lyase (1K4Y). (F) Set of *T* and *O* distributions for the chameleon section in membrane protein (4HYT). Blue dots on *X*-axis in (A, C)—chameleon fragment.

The 1K4Y and 4HYT pair is a juxtaposition of liver carboxylesterase and sodium pump subunit α-1, respectively. The chameleon fragment has a remote location with respect to the active center of the enzyme; nevertheless, the mobility (a decrease in micelle-like ordering suggests a decrease in stability) may be important for biological activity. With such a high *RD*_FR_ value of the chameleon section, it becomes reasonable to treat its role in the enzyme in question. The opposite of the expected (*T* distribution), the *O* distribution arrangement is expressed in Figure 6.

A different status was identified in the chameleon section of the 4HYT protein, where the *T* and *O* distributions show significant similarity. The variation of hydrophobicity levels typical for helix is reproduced in the actual hydrophobicity levels, presumably favoring the stabilization of the protein in the membrane (exposure of hydrophobic residues).

Similar to the arrangement discussed herein, the status of the chameleon sections in the protein pair: Homoserine dehydrogenase (1Q7G) and *C*-*x*-*c* chemokine receptor type 1 (2LNL). No catalytic residues are present in the dehydrogenase domain analyzed.

**Figure 7:**
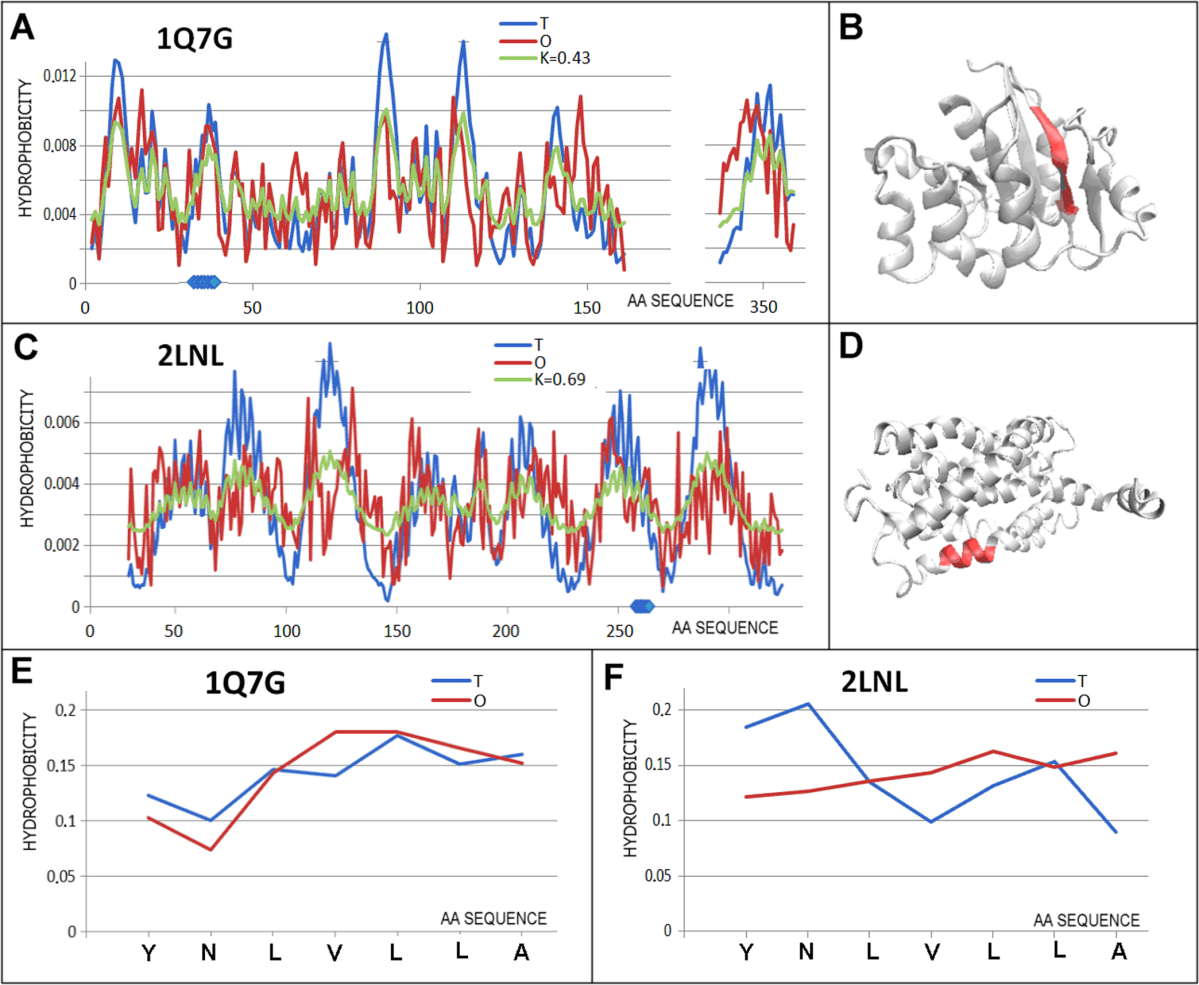
Characterization of the pair of proteins distinguished as 4 in Figure 3C. (A) *T, O*, and *M* distribution for the *K* value (given in the legend) describing the status of oxidoreductase (1Q7G). (B) 3D representation of the structural unit for which the parameters have been determined with the chameleon section (red) highlighted—1Q7G. (C) *T, O*, and *M* distribution for the *K* value (given in the legend) describing the status of protein engaged in signaling (2LNL). (D) 3D representation of the structural unit for which the parameters have been determined with the chameleon section (red) highlighted—2LNL. (E) *T* and *O* distribution for the chameleon section in oxidoreductase (1Q7G). (F) Set of *T* and *O* distributions for the chameleon section in protein engaged in signaling (2LNL). Blue dots on *X*-axis in (A, C)—chameleon fragment.

The protein pair in question shows a matching β-structure chameleon section (low *RD*_FR_ value). The characteristics of the variability in *T* levels in the chameleon section 2LNL—membrane protein—are typical, forming one highly hydrophobic side (facing the membrane) and another lower hydrophobic side involved in interactions within the domain (Figure 7). The *O*_*i*_ levels in this section represent a rather constant level of hydrophobicity, which affects the high *RD*_FR_ value expressing the state of mismatch.

Hydrophobicity levels in the chameleon section in 1Q7G follow the expected levels.

**Figure 8:**
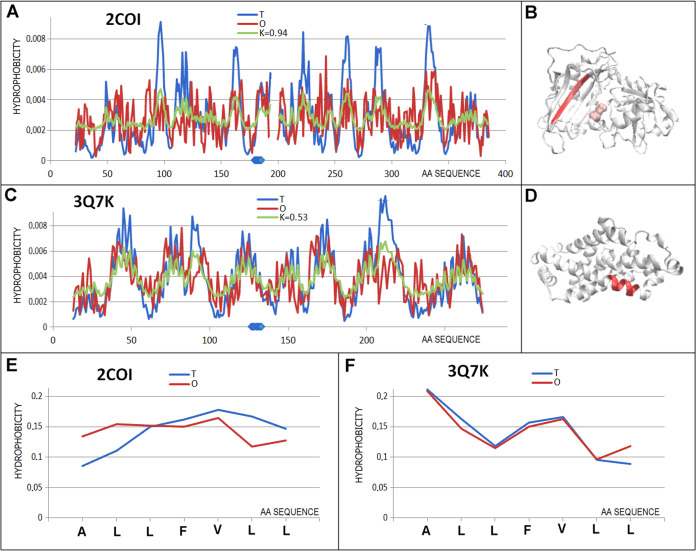
Characteristics of the pair of proteins are distinguished as 5 in Figure 3C. (A) *T, O*, and *M* distribution for the *K* value (given in the legend), describing the status of transferase (2COI). (B) 3D representation of the structural unit for which the parameters have been determined with the distinguished chameleon section (red) highlighted—2COI; residues distinguished as pink—catalytic residue. (C) *T, O*, and *M* distribution for the *K* value (given in the legend) describing the status of protein engaged in metal transport (3Q7K). (D) 3D representation of the structural unit for which the parameters have been determined with the chameleon section (red) highlighted—3Q7K. (E) *T* and *O* distribution for the chameleon section in transferase (2COI). (F) *T* and *O* distributions for the chameleon section in protein engaged in transport (3Q7K). Blue dots on the *X*-axis in (A, C)—chameleon fragment.

The pair of proteins with chameleon sections showing the highest variation is 2COI and 3Q7K (Figure 8). These proteins are transferase with one catalytic residue listed in the PDBSum base (222 K) and a transport protein–formate channel foca (3Q7K) with a significant helical structure contribution. The variation of these two proteins is already clear at the level of structural units (*K* = 0.94 and 0.55, respectively), which are both constructed with polypeptide chains (Figure 8). In contrast, chameleon sections represent a significantly different status (*RD*_FR_ for 2COI = 0.837, for 3Q7K*RD*_FR_ = 0.158). As shown in the profiles (Figure 8C,D), there is a change in the hydrophobicity levels within these sections. In the enzyme, the *O* distribution adopts a comparable level throughout the chameleon section without adjustment for the expected changes. However, when it comes to the transport protein, the *O*_*i*_ values of the entire section reproduce the expected levels of *T*_*i*_. This indicates adaptation to decreasing hydrophobicity levels starting from the N-terminal position. The adaptation seen in Figure 8D is extremely consistent (in the analysis so far, this is the lowest *RD*_FR_ value for the chain fragments analyzed). The significant variation in the status of the chameleon sections in the protein pair discussed represents the highest variation. It is the result of the adaptation of the chameleon sections to the expected task imposed on these chain sections. In this pair of proteins, the section with β-structuring shows a mismatch, while the helical fragment of membrane protein reveals a very high match of the *O* distribution with the *T* distribution, suggesting the involvement of stabilization of the hydrophobic interaction with the hydrophobic membrane.

#### Extremely High Match between the *O* Distribution and the *T* Distribution (Helical form) with Extremely High Mismatch of the Distribution in the Section Representing β-Structure (Dot 6 of Figure 3C)

**Figure 9:**
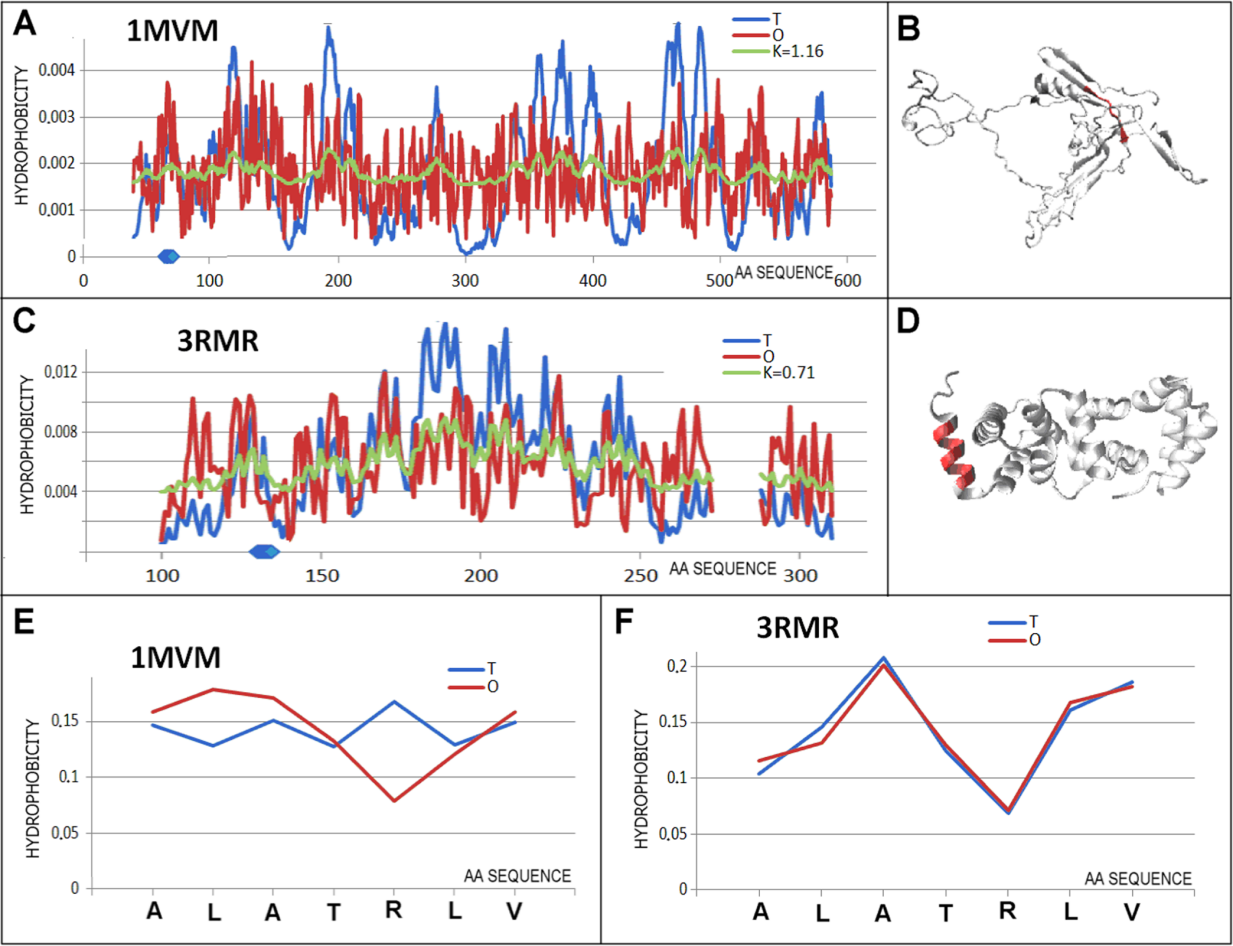
Characteristics of the pair of proteins distinguished as 6 in Figure 3C. (A) *T, O*, and *M* distribution for the *K* value (given in the legend) describing the status of DNA binding protein (1MVM). (B) 3D representation of the structural unit for which the parameters have been determined with the chameleon section (red) highlighted—1MVM. (C) *T, O*, and *M* distribution for the *K* value (given in the legend) describing the status of protein engaged in protein binding (3RMR). (D) 3D representation of the structural unit for which the parameters have been determined with the chameleon section (red) highlighted—3RMR. (E) *T* and *O* distribution for the chameleon section in a DNA binding protein (1MVM). (F) Set of *T* and *O* distributions for the chameleon section in protein binding (3RMR). Blue dots on *X*-axis in (A, C)—chameleon fragment.

1MVM-A (549 aa) β DNA binding virus protein—β-structure ALATRLV 3RMR—protein binding—avirulence protein ATR1 (236 aa)—helix (Figure 9).

The present pair of chameleon proteins is characterized by extreme values describing the status of the chameleon section. The status of the chameleon section present in 1MVM is expressed with the highest value *K* = 1.16 while the status of the chameleon section in the 3RMR protein is expressed with the lowest value (*RD*_FR_ = 0.040).

#### Comparable Status of Chameleon Sections at a Very High Match of the Helical Form (Dot 7 Figure 3C)

**Figure 10:**
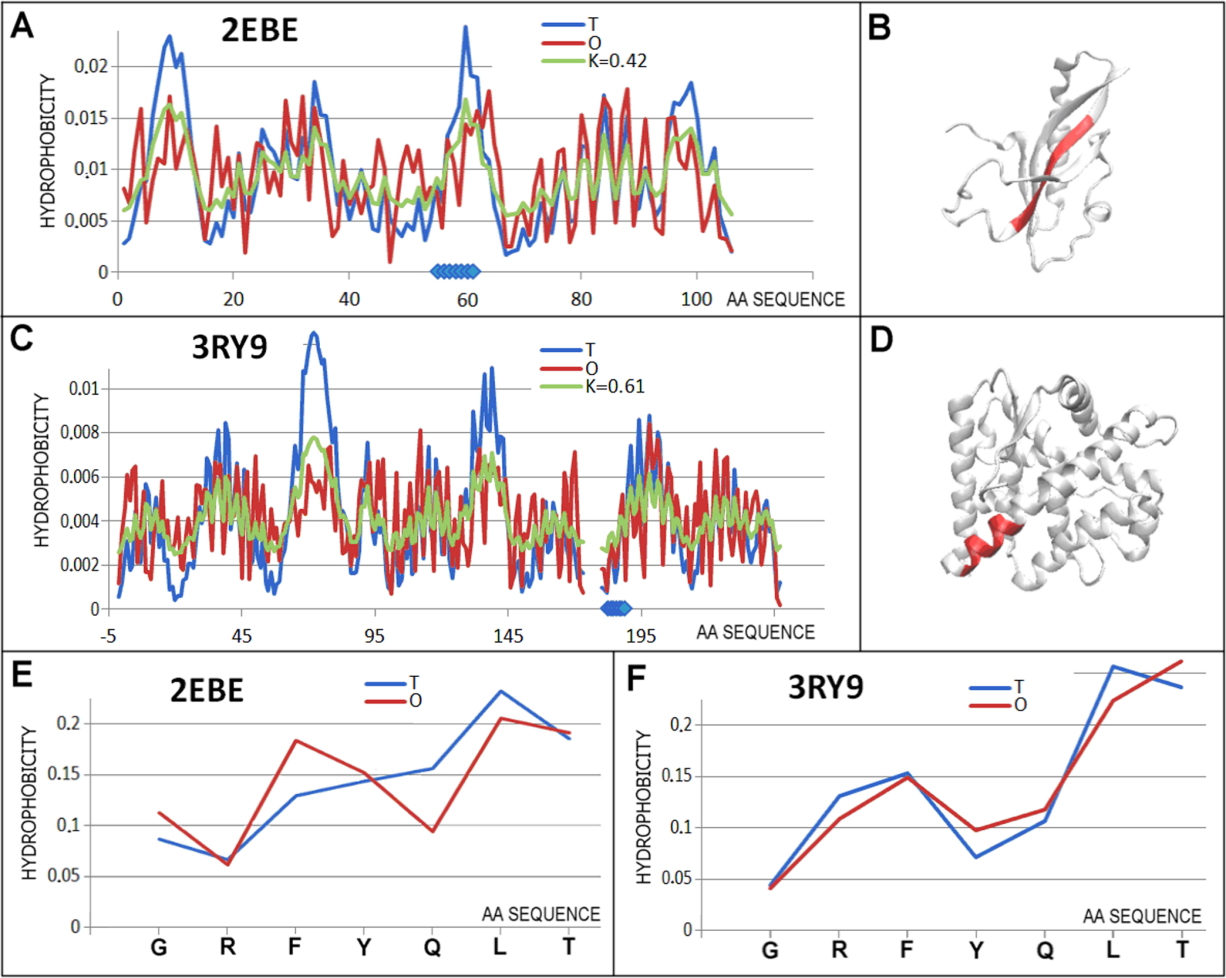
Characteristics of the pair of proteins are distinguished as 7 in Figure 3C. (A) *T, O*, and *M* distribution for the *K* value (given in the legend) describing the status of protein of unknown activity (2EBE). (B) 3D representation of the structural unit for which the parameters have been determined with the chameleon section (red) highlighted—2EBE. (C) *T, O*, and *M* distribution for the *K* value (given in the legend) describing the status of protein engaged in steroid binding (3RY9). (D) 3D representation of the structural unit for which the parameters have been determined with the chameleon section (red) highlighted—3RY9. (E) *T* and *O* distributions for the chameleon section in the 2EBE protein of unknown activity. (F) Set of *T* and *O* distributions for the chameleon section in steroid binding protein 3RY9. Blue dots on *X*-axis in (A, C)—chameleon fragment.

A comparable status of chameleon sections is represented by the 2EBE and 3RY9 proteins. The 2EBE protein represents an unrecognized biological function (Figure 9). Source organism *Thermus thermophilus* makes it unique, however, due to external conditions far from organisms living in conditions thermally far from extreme conditions. Both proteins: Steroid binding protein (Ancestral glucocorticoid receptor 1) synthetic source (3RY9) together with the thermophilic protein of yet unknown biological function discussed here (2EBE) fall into the group defined by the criterion *RD* > 0.5, exceed this threshold by a relatively little value (0.508 and 0.571, respectively), contain in their structure chameleon sections with a status expressed by very low *RD*_FR_ values (0.308 and 0.078, respectively), indicating a high match of the *O* distribution with the *T* distribution with this match in the case of 3RY9 being extremely low (profile set Figure 10D). Despite this difference in the *RD*_FR_ values for the chameleon sections, this protein pair, in a qualitative assessment, can be qualified as showing a jointly high match of the status of the chameleon sections with the expected state predicted in the assessment of the relationship between the *O* distribution to the *T* distribution.

#### Surprising Extreme Match of Chameleon Sections in Proteins with a High Degree of Micelle-like Disorder (Dot 8 in Figure 3C)

**Figure 11:**
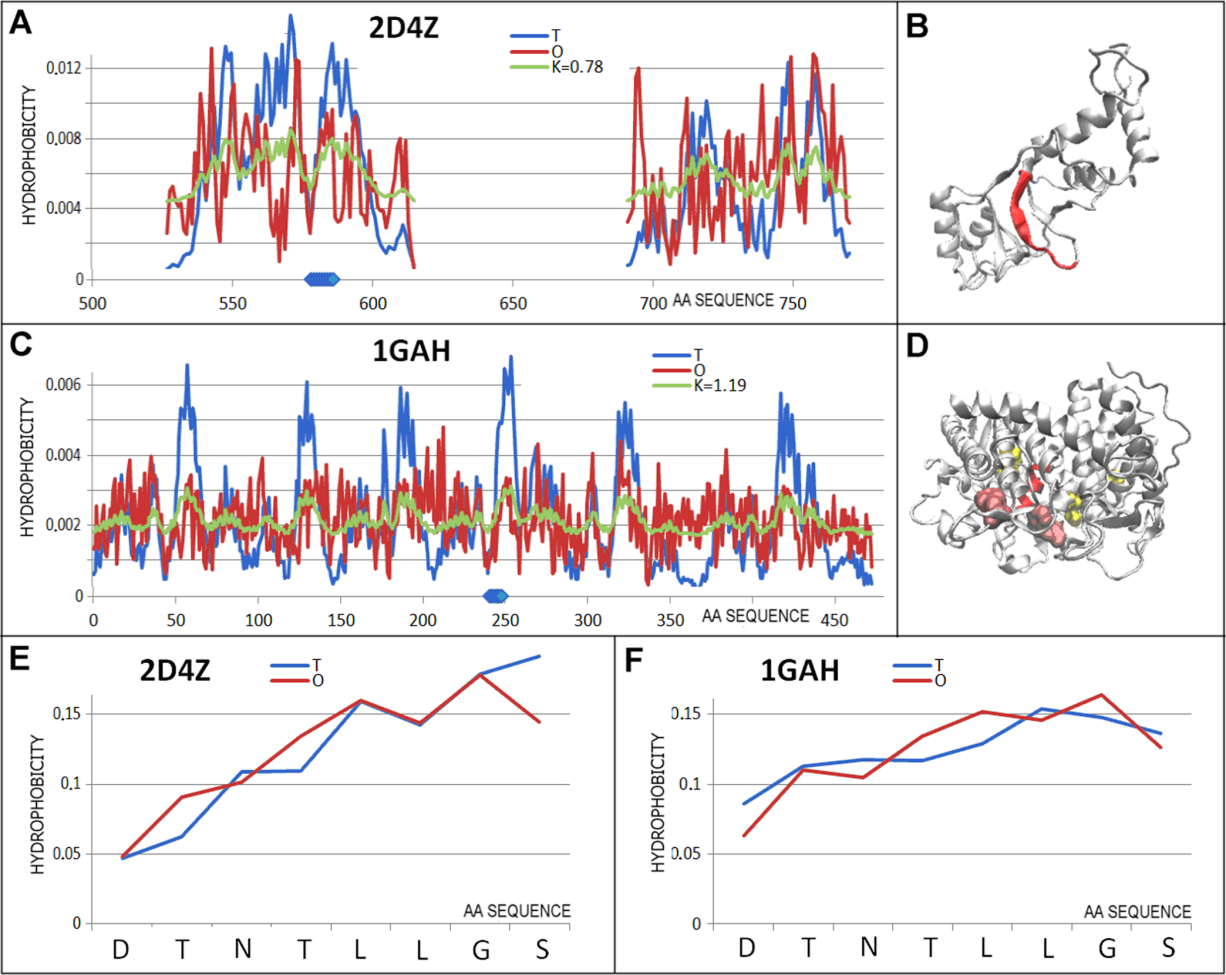
Characteristics of the pair of proteins are distinguished as 8 in Figure 3C. (A) *T, O*, and *M* distributions for *K* value (given in the legend) describing the status of protein engaged in transport (2D4Z). (B) 3D representation of the structural unit for which the parameters have been determined with the chameleon section (red) highlighted—2D4Z. (C) *T, O*, and *M* distribution for the *K* value (given in the legend), describing the status of hydrolase (1GAH-A) (D) 3D representation of the structural unit for which the parameters have been determined with the chameleon section (red) highlighted—1GAH. Additionally, the positions of the catalytic residues are highlighted in pink. (E) *T* and *O* distributions for the chameleon section in protein engaged in protein binding—2D4Z. (F) Set of *T* and *O* distributions for the chameleon section in hydrolase 1GAH. Blue dots on *X*-axis in (A, C)—chameleon fragment.

An opposite example to the previous one is the juxtaposition of 2D4Z and 1GAH. This example is different from the others because of the higher number of amino acids in the chameleon section—8 aa (Figure 11).

This pair of chameleon proteins represents the status of a structural unit with *RD* > 0.5, similarly to the entire selected subgroup of chameleon proteins. The chameleon sections, on the other hand, in both cases represent an extremely high match of a micelle-like distribution.

The 2D4Z-A protein is a transport protein (the chain contains 169 amino acids) with a chameleon section with DTNTLLGS sequence. In contrast, 1GAH-A is an enzyme (E.C.3.2.1.3-glucan 1,4-α-glucosidase 471 aa), where the complete chain shows a status with *RD* > 0.5.

The two compared proteins differ in chain length, biological function, and the proportion of secondary structure in their construction: 2D4Z has a structure composed of both helical fragments with a β plate present in the central part of the protein. In contrast, the structure of the enzyme discussed shows a predominant contribution of helical structures. Both proteins show a significant deviation from a micelle-like arrangement. The *RD* parameter values for the proteins are 2D4Z – 0.670 (*K* = 0.78) and for 1GAH – 0.734 (*K* = 1.19) (Figure 11).

In contrast, the chameleon sections represent, in these structures that are highly mismatched with a micelle-like arrangement, a locally near-perfect match between the O distribution and the T distribution expressed by very low values of *RD*_FR_ = 0.195 and *RD*_FR_ = 0.204 for 2D4Z and 1GAH, respectively. This is illustrated in Figure 11C,D.

The match of the chameleon section located in the central part of the structure in close proximity to the catalytic residues most probably provides a suitable local external field for the catalytic center. Here, the environment for the catalytic reaction appears to be different from that of the example previously discussed (2Z1K).

## Discussion

The experimentally identified match of the secondary structure form dominating in a protein due to the introduction of a single mutation with preservation in both status forms of the micelle-like hydrophobicity arrangement proves the validity of the hypothesis.^42−45^ In the proteins discussed in these papers, it appears to be paramount to achieve a micelle-like status with a centrally located hydrophobic core against secondary structure forms that achieve this type of distribution.^46−53^ These papers demonstrate the pursuit of appropriate structuring dominated by a specific secondary structure form caused by the introduction of a single-amino-acid mutation. This pursuit of appropriate structuring in the cited proteins means achieving micelle-like structuring.

The sub-base analyzed herein represents proteins with a status of structural units representing a hydrophobicity system different from micelle-like (*RD* > 0.5). Such a status is interpreted as being achieved with the support of variable external factors different from polar water (external force field). Such an influence is shown, for example, by the immediate vicinity of the cell membrane (membrane proteins represented in this analysis)^10^ and other factors such as chaperone.^38^ Factors disturbing the specificity of the polar water environment can be very diverse and thus unpredictable. However, the proteins discussed in the present analysis represent a relatively consistent and comparable status identified for pairs of chameleon sections. With the unpredictability of environmental conditions, a high variation in the status of chameleon sections can be predicted especially if the helical ordering relationship with β-structure is analyzed. However, it turns out that these sections show a highly comparable status regardless of the status of the structural unit in which the sections are included. Obtaining a comparable status of sections with a different secondary structure in proteins with a variable biological function (Table 2) reveals the importance of secondary structure as a secondary factor leading to the appropriate status (hydrophobicity distribution) expected from the point of view of the ordering of the structural unit as a whole. The form of secondary structure thus acts as a means to achieving the appropriate status in relation to the structural unit. Secondary structure is not an end in itself. It is a means of achieving the goal set for the structural unit as a whole.

In the analysis of the dependence of protein structuring on environmental conditions, an emphasis is placed on the transformation leading to the formation of the amyloid form dominated by the β-structure form.^52−57^ Proteins with available structures of both native and amyloid forms are particularly important in this analysis: α-synuclein, V-domain of light chain of IgG, and transthyretin. Using the FOD-M model to analyze these structural changes, the role of the environment directing a different ordering depending on the specificity of the local environment is revealed.^10^ The analysis of amyloid structures reveals the influence of environmental conditions on the folding process.^58−60^

## Conclusions

The postulate of nonlocal targeting vs broader context-dependent adoption of the appropriate form of secondary structure, referred to as context-dependent, was presented in refs (58−60). This means that folding depends on the amino acid sequence but also on other factors, including environmental factors in particular. Depending on the directing contribution of the environment of a folding protein, fragments of chains with an identical sequence can acquire different secondary forms.

The present study supports the role of hydrophobicity distribution in determining the tertiary structure of the entire protein molecule. Converging upon a confirmation that is compatible with the local environment appears to outweigh the conformational preferences of individual fragments. Thus, secondary folds seem to be a means to an end, i.e., shaping the structure of the protein as a whole, rather than a goal unto itself. According to the presented hypothesis, the secondary structural characteristics of individual fragments align with the overall distribution of hydrophobicity, which describes the tertiary structure of the protein. The role of the environment is also highlighted by the analysis of the amyloid transformation process, where the importance of external conditions—external force field directing the folding process—was demonstrated.^51−54^ The finding that any chain can transform into amyloid under specific conditions emphasizes the importance of environmental influences on protein structuring.^57−60^ This phenomenon has also been identified based on the fuzzy oil drop model.^51−54^ The group of proteins analyzed in the present work is characterized by a structure directed by the local environment, which can theoretically be very different. Nevertheless, the vast majority of the chameleon section structuring favors the ordering resulting from the direction of the external force field.^10^
